# Unusual Abdominal Wall Complication After a Bilateral Transversus Abdominis Plane Block for Postoperative Analgesia

**DOI:** 10.7759/cureus.104399

**Published:** 2026-02-27

**Authors:** Ajay Pius, Bassam Durgham, Stephen Vanbeek, Estella Fye

**Affiliations:** 1 Anesthesiology, Henry Ford St. John Hospital, Detroit, USA

**Keywords:** abdominal wall ulceration, autoinflammatory dermatosis, local anesthetic, neutrophilic dermatosis, pathergy reaction, postoperative complication, pyoderma gangrenosum, regional anesthesia, tap block, wound ulceration

## Abstract

Transversus abdominis plane (TAP) blocks are widely regarded as safe techniques for postoperative analgesia. We describe an unusual postoperative complication characterized by rapidly progressive bilateral abdominal wall ulceration following bilateral TAP block and infiltration of laparoscopic port sites with local anesthetics. The multifocal distribution, poor response to antibiotics, negative cultures, and association with procedural trauma raised strong concern for a sterile autoinflammatory dermatosis consistent with pyoderma gangrenosum or a related neutrophilic process, although a definitive diagnosis could not be confirmed. While pathergy remains the most widely accepted trigger for such reactions, an immune-mediated response to local anesthetic exposure may have contributed. The patient ultimately required surgical debridement with subsequent improvement. This case highlights the importance of recognizing noninfectious autoinflammatory wound reactions following regional anesthesia.

## Introduction

Transversus abdominis plane (TAP) blocks are frequently used to provide somatic analgesia to the anterior abdominal wall following abdominal surgery [1,2]. Complications are uncommon and typically limited to hematoma, infection, or inadvertent peritoneal injury [1,2]. Sterile autoinflammatory dermatoses such as pyoderma gangrenosum have been described as postoperative complications and may be triggered by minor trauma through pathergy mechanisms [3-6]. These conditions can progress rapidly, are often initially misdiagnosed as infection, and may worsen with surgical manipulation [3-7]. We describe a case of progressive bilateral abdominal wall ulceration following TAP block and local anesthetic infiltration of laparoscopic port sites. The clinical features raised concern for a sterile autoinflammatory neutrophilic dermatosis within the pyoderma gangrenosum spectrum, possibly facilitated by both procedural trauma and immune-mediated responses to local anesthetic exposure. 

## Case presentation

A 19-year-old female weighing 55 kg with a history of recurrent small bowel obstruction secondary to prior abdominal surgery presented for elective diagnostic laparoscopy due to persistent obstructive symptoms. The patient had no history of inflammatory bowel disease, autoimmune conditions, dermatologic disorders, coagulopathy, or immunodeficiency. There was no personal or family history of neutrophilic dermatoses. The patient was not taking anticoagulants or immunosuppressive medications and had no known drug allergies.

Intraoperatively, adhesive disease was identified, and adhesiolysis was performed without bowel injury or ischemia. Estimated blood loss was minimal, and the patient remained hemodynamically stable throughout.

For postoperative analgesia, bilateral ultrasound-guided TAP blocks were performed using sterile technique and a high-frequency linear probe. After negative aspiration, 30 mL of 0.2% ropivacaine was injected into the fascial plane on each side without resistance or signs of intravascular injection. No immediate complications occurred. Prior to skin closure, laparoscopic port sites were infiltrated by the surgical team with 5 mL of 0.25% bupivacaine as an additional analgesic measure. The cumulative local anesthetic dose administered intraoperatively remained within recommended weight-based safety limits.

The immediate postoperative course was uncomplicated, with stable vital signs and satisfactory pain control.

**Figure 1:**
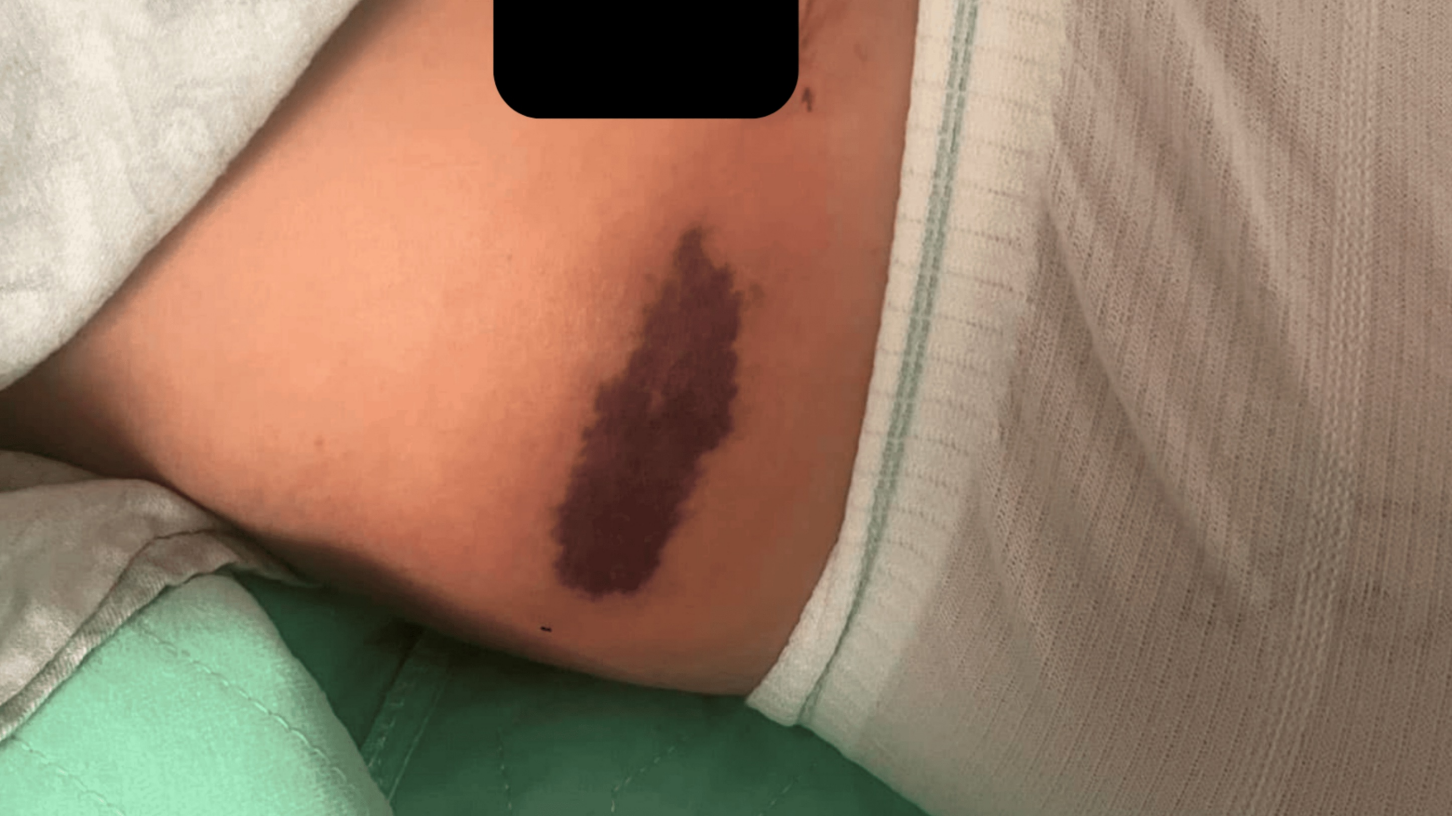
Postoperative day 1 demonstrating marked ecchymosis over right lateral abdominal wall at the transversus abdominis plane (TAP) block site.

On postoperative day (POD) 1, the patient developed localized ecchymosis and tenderness over the right lateral abdominal wall corresponding to the TAP block site (Figure 1).

The patient was afebrile and hemodynamically stable. Laboratory evaluation demonstrated a normal white blood cell count, stable hemoglobin, normal platelet count, and normal coagulation studies.

**Figure 2:**
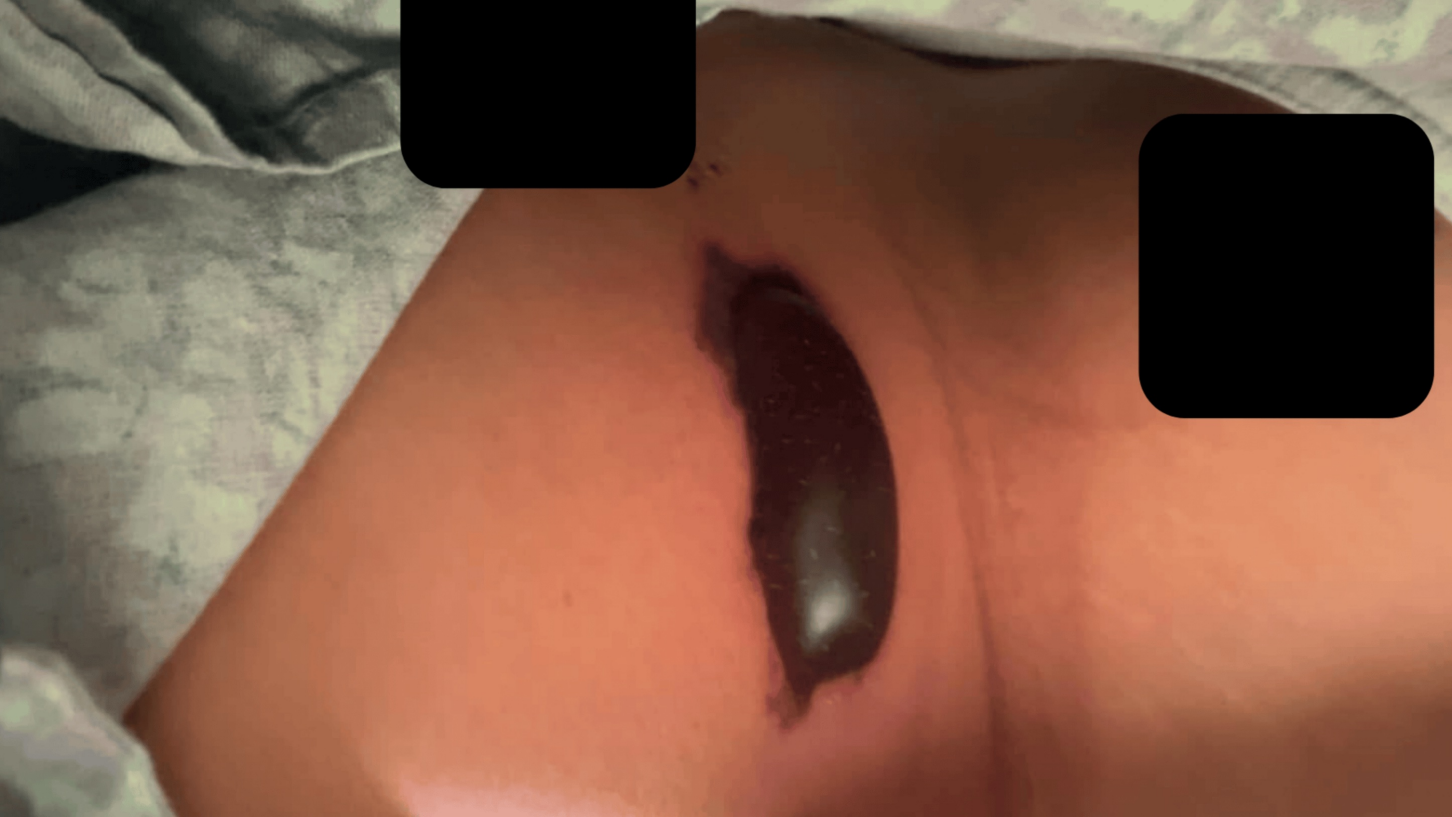
Postoperative day 2 demonstrating blistering and violaceous discoloration involving bilateral abdominal wall and port sites.

By POD 2, the affected area progressed to blistering and violaceous discoloration with irregular borders (Figure 2).

Similar but less extensive lesions were observed over the contralateral TAP site and several laparoscopic port incisions. The lesions were painful but without fluctuance, crepitus, or purulent drainage. Given concern for cellulitis, the general surgery team initiated empiric intravenous vancomycin and obtained an infectious disease consultation. Despite three days of antimicrobial therapy, the patient remained afebrile without leukocytosis and demonstrated continued lesion progression. Antibiotics were subsequently discontinued.

Computed tomography of the abdomen and pelvis demonstrated inflammatory changes limited to the superficial abdominal wall without abscess, organized fluid collection, hematoma, fascial gas, or evidence of deep soft tissue involvement [8]. There was no evidence of intra-abdominal pathology.

**Figure 3:**
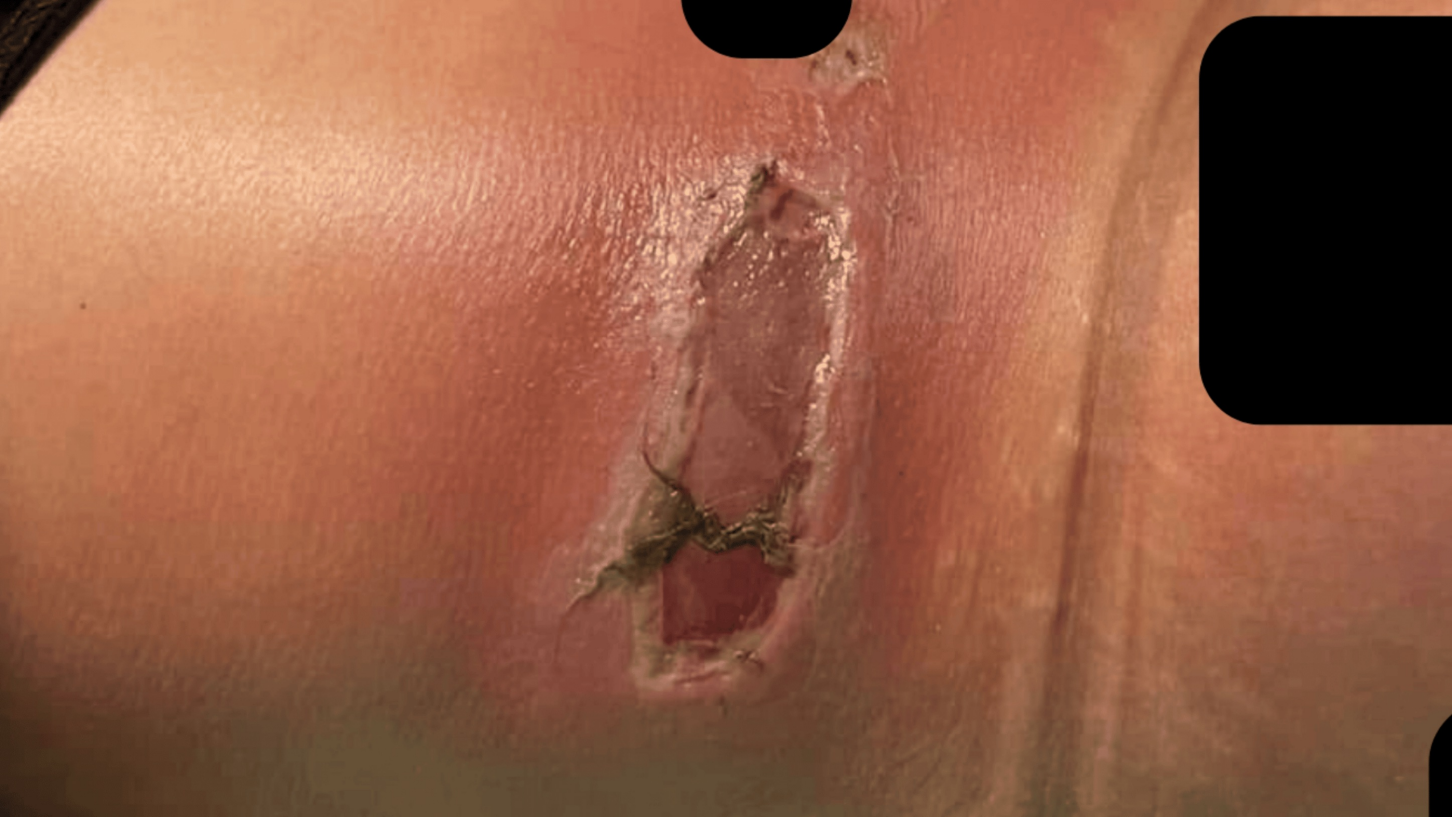
Postoperative day 5 demonstrating ulceration with tissue sloughing prior to surgical debridement.

Despite antimicrobial therapy, the lesions continued to enlarge over the next 48 hours. By POD 5, ulceration with tissue sloughing and undermined borders had developed at multiple sites (Figure 3).

The multifocal distribution involved both TAP injection sites and laparoscopic port incisions. The patient remained afebrile. Blood cultures and wound cultures demonstrated no microbial growth.

Given progression despite antibiotics and absence of radiographic or laboratory evidence of necrotizing infection, surgical exploration was undertaken to exclude deep soft tissue infection. Operative findings revealed superficial tissue necrosis without fascial or deep muscular involvement and no purulence. Only the largest ulcerative lesion was surgically debrided and sent for culture and histopathologic evaluation. Histopathologic examination demonstrated a dense neutrophilic inflammatory infiltrate with mixed acute and chronic inflammation. No organisms were identified on Gram stain or special stains, and there was no evidence of vasculitis, thrombosis, or malignancy.

The patient was managed with local wound care. Over subsequent days, the lesions stabilized and gradually improved with re-epithelialization. No additional surgical intervention was required. The patient was discharged in stable condition with outpatient wound follow-up.

Written informed consent for publication of clinical details and images was obtained.

## Discussion

This case represents an unusual postoperative wound complication following a TAP block and local infiltration of local anesthetic. The bilateral involvement, multifocal distribution, rapid progression, culture negativity, absence of organized fluid collection on imaging, and limited response to antibiotic therapy are not consistent with infectious cellulitis, ischemic necrosis, or dose-dependent chemical toxicity [9,10]. The toxic effects of local anesthetics tend to be localized and would not be expected to involve noncontiguous sites such as laparoscopic port incisions [10].

The clinical presentation raised concern for a sterile autoinflammatory neutrophilic dermatosis consistent with pyoderma gangrenosum [3-7]. Pyoderma gangrenosum has been described as a postoperative complication and may be precipitated by minor trauma through pathergy [4-7]. Pathergy refers to a phenomenon in which seemingly minor mechanical injury, such as needle insertion or surgical incision, triggers a disproportionately intense inflammatory response leading to progressive tissue breakdown. This reaction is thought to reflect dysregulated neutrophilic activity and aberrant innate immune signaling rather than infection. Clinically, lesions may worsen following debridement or additional procedural intervention. Pyoderma gangrenosum is frequently misdiagnosed as an infection, often progresses despite antibiotic therapy, and may deteriorate with surgical manipulation [3-7]. Although classically associated with inflammatory bowel disease, hematologic malignancy, and systemic inflammatory disorders, many patients lack an identifiable underlying condition [6,7].

While procedural trauma remains the most widely recognized initiating mechanism, an immune-mediated response to local anesthetic exposure may have contributed. Local anesthetics have been shown to influence neutrophil chemotaxis, cytokine signaling, endothelial activation, and wound healing responses in vitro [11-13]. These immune effects may amplify sterile inflammatory responses in susceptible patients. In this case, both ropivacaine and bupivacaine were administered at separate anatomical sites that later developed similar ulcerative changes, supporting a mechanism beyond focal toxicity. The continuation of antibiotics despite negative infectious evaluation and lack of therapeutic response further argues against infection as the primary process. Earlier involvement of dermatology may have facilitated more rapid recognition of a sterile neutrophilic dermatosis and potentially reduced unnecessary antibiotic exposure in this case.

## Conclusions

TAP blocks are generally safe regional anesthetic techniques; however, sterile autoinflammatory wound reactions may rarely occur in the postoperative setting. Multifocal ulcerative lesions that are unresponsive to antibiotics and accompanied by negative infectious evaluation should prompt consideration of neutrophilic dermatoses including pyoderma gangrenosum. Recognition of such conditions is important to avoid unnecessary antimicrobial exposure and delays in appropriate management. Further investigation is needed to better understand interactions between local anesthetic exposure, procedural trauma, and autoinflammatory wound responses. 
